# Gender and Ethnic Disparities of Acute Kidney Injury in COVID-19 Infected Patients: A Literature Review

**DOI:** 10.3389/fcimb.2021.778636

**Published:** 2022-01-13

**Authors:** Weihang He, Xiaoqiang Liu, Bing Hu, Dongshui Li, Luyao Chen, Yu Li, Ke Zhu, Yechao Tu, Situ Xiong, Gongxian Wang, Bin Fu

**Affiliations:** ^1^ Reproductive Medicine Center, The First Affiliated Hospital of Nanchang University, Nanchang, China; ^2^ Department of Urology, The First Affiliated Hospital of Nanchang University, Nanchang, China; ^3^ Jiangxi Institute of Urology, Nanchang, China

**Keywords:** SARS-CoV-2, COVID-19, ACE2, acute kidney injury, gender differences, ethnic differences

## Abstract

Coronavirus disease 2019(COVID-19) has become a public health emergency of concern worldwide. COVID-19 is a new infectious disease arising from Coronavirus 2 (SARS-CoV-2). It has a strong transmission capacity and can cause severe and even fatal respiratory diseases. It can also affect other organs such as the heart, kidneys and digestive tract. Clinical evidence indicates that kidney injury is a common complication of COVID-19, and acute kidney injury (AKI) may even occur in severely ill patients. Data from China and the United States showed that male sex, Black race, the elderly, chronic kidney disease, diabetes, hypertension, cardiovascular disease, and higher body mass index are associated with COVID-19‐induced AKI. In this review, we found gender and ethnic differences in the occurrence and development of AKI in patients with COVID-19 through literature search and analysis. By summarizing the mechanism of gender and ethnic differences in AKI among patients with COVID-19, we found that male and Black race have more progress to COVID-19-induced AKI than their counterparts.

## Introduction

Coronavirus disease 2019 (COVID-19) is an infectious disease caused by the infection of severe acute respiratory syndrome coronavirus 2 (SARS-CoV-2). As of May 23, 2021, there were 167 million confirmed cases and 3,475,086 deaths worldwide (https://www.worldometers.info/coronavirus/). SARS-CoV-2 uses the host cell’s angiotensin-converting enzyme 2 (ACE2) as a receptor to invade cells, thereby affecting their normal physiological functions. As the “gateway” recognized by SARS-CoV-2, ACE2 is not only expressed in lung cells, but also in podocytes and proximal tubules of the kidney. After binding to ACE2, the spike protein of SARS-CoV-2 is hydrolyzed and cleaved by type II transmembrane serine protease (TMPRSS2), which promotes the virus to invade host cells (Hoffmann et al., 2020; Matsuyama et al., 2020). Clinical evidence also shows that kidney involvement is common during the course of COVID-19, and the incidence of acute kidney injury (AKI) in severe COVID-19 patients is also high (Nadim et al., 2020). Current evidence suggests that AKI caused by COVID-19 may affect > 50% of patients in the ICU (Nadim et al., 2020). In addition, a consistent feature of the COVID-19 pandemic is that men are susceptible and have poor outcomes (Naicker et al., 2020). In several countries, men account for the majority of COVID-19 deaths. In China, men accounted for 73% of deaths, 59% in South Korea, and in Italy, 70% of deaths are men (La Vignera et al., 2020). Data from China and the United States showed that male sex, Black race, the elderly, chronic kidney disease (CKD), diabetes, hypertension, cardiovascular disease, and higher body mass index are associated with COVID-19‐induced AKI (Hirsch et al., 2020; Pei et al., 2020). These findings indicate the presence of an association between males and higher mortality. It is known that AKI is an indicator of negative prognosis and disease severity in patients with COVID-19 (Nadim et al., 2020; Varga et al., 2020). However, currently few reports have focused on gender differences in COVID-19 patients with kidney injury. By summarizing the relevant research data published recently, this review not only found gender differences in AKI caused by COVID-19, but also observed ethnic differences in the occurrence and progression of AKI. And by describing and discussing the mechanism of these two differences, it helps to design better prevention and therapeutic strategies.

## Gender Differences in the Incidence of AKI Among Patients With COVID-19

**Figure 1:**
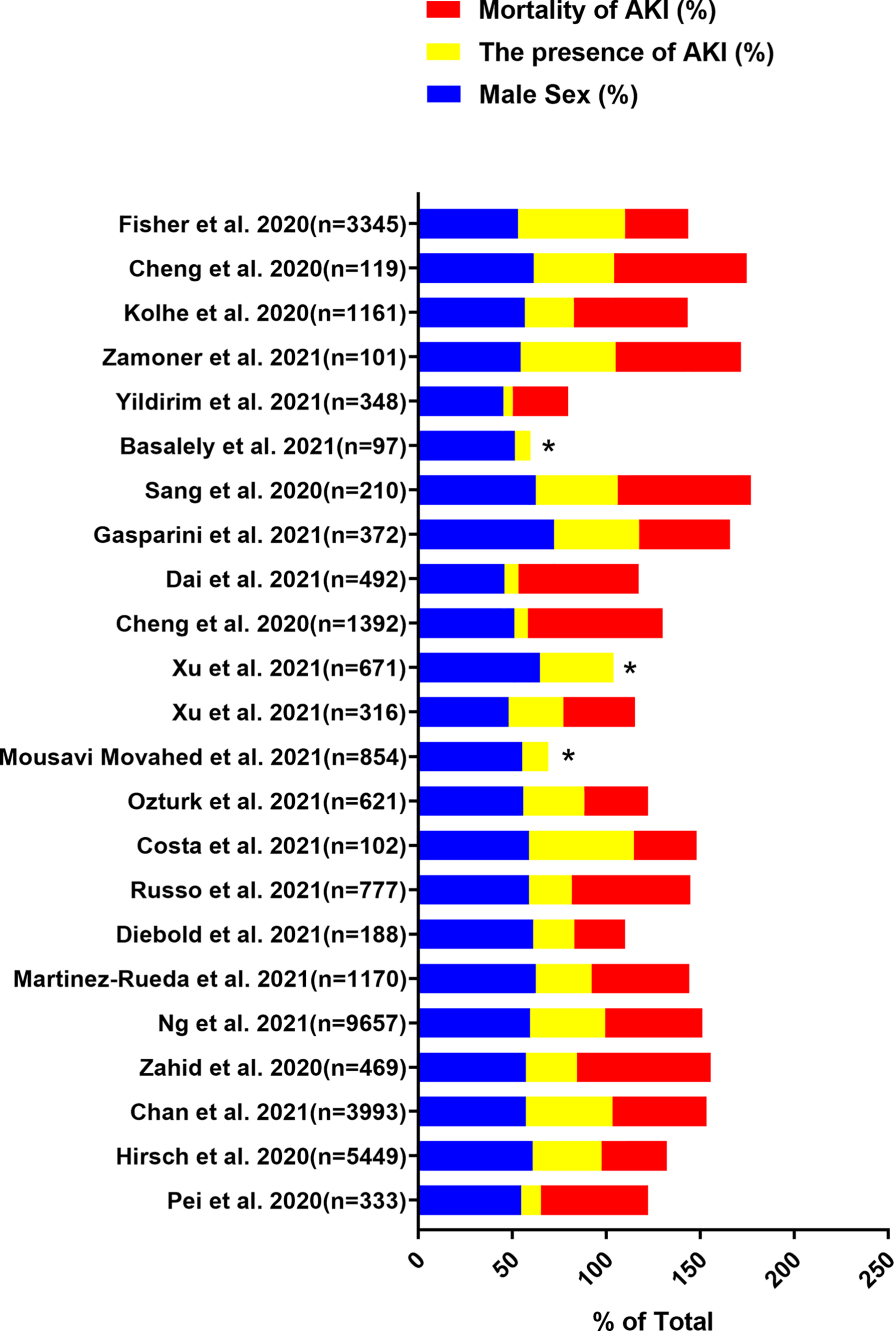
Data are extracted from 23 studies on COVID-19 patients, including the total number of patients included, the proportion of male patients, the incidence of AKI and the mortality of AKI. ^“*”^: The mortality of AKI is unavailable.

**Figure 2:**
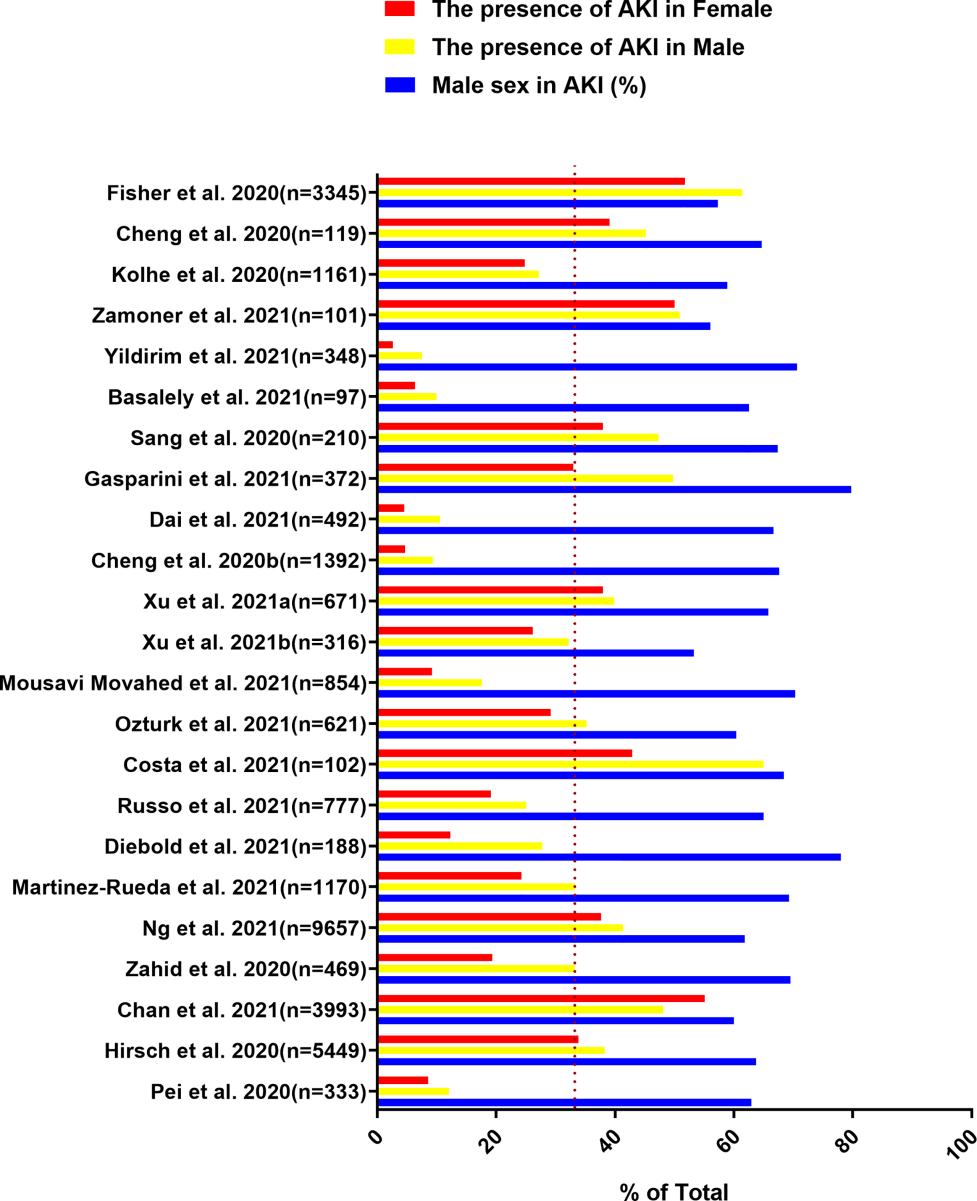
Data are extracted from 23 studies on COVID-19 patients, including the total number of patients included, the proportion of males in AKI patients, the incidence of AKI in male patients and the incidence of AKI in female patients.

The gender-related COVID-19 mortality rate is one of the most frequently reported epidemiological data. Current evidence indicates that the incidence rate is higher in males than in females. In addition, males show more serious results than females (Mauvais-Jarvis, 2020; Peckham et al., 2020). Data of 59,254 patients from 11 different countries showed an association between males and high mortality (Borges do Nascimento et al., 2020). We identified the data by searching PubMed and references from relevant articles using the search terms “Coronavirus Disease 2019”, “COVID-19”, “SARS-CoV-2”, “kidney disease”, “acute kidney injury”, “AKI”, “risk factors”, “gender”, “clinical outcomes”, and “Clinical Characteristics”. We found that the SARS-CoV-2 infection rate in males is higher than that in females, and the incidence of AKI in males is also higher than that in females in most studies (**Figures 1**, **2**) (Cheng et al., 2020b; Fisher et al., 2020; Hirsch et al., 2020; Kolhe et al., 2020; Pei et al., 2020; Sang et al., 2020; Zahid et al., 2020; Basalely et al., 2021; Chan et al., 2021; Cheng et al., 2021; Costa et al., 2021; Dai et al., 2021; Diebold et al., 2021; Gasparini et al., 2021; Martinez-Rueda et al., 2021; Mousavi Movahed et al., 2021; Ng et al., 2021; Ozturk et al., 2021; Russo et al., 2021; Xu H. et al., 2021; Xu J. et al., 2021; Yildirim et al., 2021; Zamoner et al., 2021). In these studies, all of the patients with COVID-19 had new-onset AKI during hospitalization. The COVID-19 mortality rate is related to comorbidities. The latest data suggest that male sex, older age, CKD, diabetes, hypertension, cardiovascular disease, obesity, genetic risk factors, immunosuppression, and smoking history may induce or increase the incidence and progression of AKI (Hirsch et al., 2020; Nadim et al., 2020; Pei et al., 2020). Recent studies have also shown that the male sex is an independent predictor for AKI in patients with COVID-19 (Hirsch et al., 2020). This may be related to the higher infection rate of males with SARS-CoV-2.

Mechanically, compared with females, males are more susceptible to viral infections because of the different innate immunities related to sex chromosomes (Conti and Younes, 2020). Males experience infectious diseases more frequently and with increased severity (Crommelin, 2013), but females are more prone to the majority of autoimmune diseases (Beeson, 1994; Whitacre et al., 1999). Furthermore, ACE2 and TMPRSS2, as the key factors for virus invasion (Shang et al., 2020), are more highly expressed in males than females. Recent studies have shown that androgens (AR) regulate the expression of ACE2 and TMPRSS2, including the lungs, which may explain the increased susceptibility of males to severe SARS-CoV-2 infection (Mikkonen et al., 2010; Sama et al., 2020). AR can also modulate the immune response and reduce the antibody response to viral infection to increase the severity of SARS-CoV-2 infection in males (Klein and Flanagan, 2016). Interestingly, estradiol treatment can significantly reduce ACE2 mRNA levels in normal human bronchial epithelial cells (Stelzig et al., 2020). In addition, a study found that estradiol and phytoestrogens genistein can reduce the mRNA levels of TMPRSS2, indicating that estrogen may play a protective role in COVID-19 infection by inhibiting SARS-CoV-2 from entering host cells (O’Brien et al., 2020). Therefore, gender differences in hormone levels may contribute to male susceptibility to SARS-CoV-2. Moreover, the presence of underlying diseases such as CKD, diabetes, hypertension, and cardiovascular diseases will increase the infection rate and mortality of SARS-CoV-2. Data from the Public Health Agency of Canada shows that males are more likely to suffer from diabetes and heart disease than females (O’Brien et al., 2020). Evidence from epidemiological observations showed that diabetes patients with COVID-19 have a 50% increased risk of fatal outcomes compared with patients without diabetes (Zhu et al., 2020). Single-cell RNA analysis has shown that male, old age, and smoking habits can increase the expression of ACE2 and TMPRESS2 (Muus et al., 2021), thereby promoting SARS-CoV-2 infection. These gender differences in behavior may also increase the risk of males suffering from underlying diseases, which provides a possible explanation for the higher infection rate in males (Ellison and Tasian, 2021). Finally, gender-related susceptibility may also be related to vitamin D. Vitamin D deficiency is an independent risk factor for viral acute respiratory infection (ARI). A study showed that men have lower vitamin D supplements compared with age-matched females (La Vignera et al., 2020). This is also a factor that leads to gender differences in susceptibility.

In addition to the higher proportion of males in AKI patients, the incidence of AKI in male patients with COVID-19 is also higher than that in female patients (**Figure 2**) (Cheng et al., 2020b; Fisher et al., 2020; Hirsch et al., 2020; Kolhe et al., 2020; Pei et al., 2020; Sang et al., 2020; Zahid et al., 2020; Basalely et al., 2021; Chan et al., 2021; Cheng et al., 2021; Costa et al., 2021; Dai et al., 2021; Diebold et al., 2021; Gasparini et al., 2021; Martinez-Rueda et al., 2021; Mousavi Movahed et al., 2021; Ng et al., 2021; Ozturk et al., 2021; Russo et al., 2021; Xu H. et al., 2021; Xu J. et al., 2021; Yildirim et al., 2021; Zamoner et al., 2021). According to the previously mentioned mechanism of SARS-CoV-2 directly invading kidney host cells to cause AKI, ACE2 and TMPRSS2 are expressed in proximal convoluted tubule cells and podocytes (He et al., 2020). Therefore, it is important to understand the gender differences in the expression of ACE2 and TMPRSS2 in the kidney. A study pointed out that AR could upregulate the expression of ACE2 in human glomerular epithelial cells, tubular cells and podocytes (Yanes Cardozo et al., 2021), providing a theoretical basis for the high incidence of AKI in males. Furthermore, AR could increase the efferent arteriolar resistance by increasing the level of Ang II, thereby exacerbating glomerular injury (Reckelhoff et al., 2005). The transcription of TMPRSS2 is also regulated by AR (Lucas et al., 2014). Thus, AR will not only increase the risk of SARS-CoV-2 kidney infection, but also cause aggravation of kidney damage. AKI caused by SARS-CoV-2 infection has many indirect factors, such as dysregulated immune responses, cytokine storm, endothelial dysfunction, and hypercoagulability. These indirect factors also have gender differences. Dysregulated immune responses can be observed in patients with severe COVID-19, and the neutrophils, leukocytes, and neutrophil to lymphocyte ratio (NLR) are significantly increased in these patients (Qin C.et al., 2020; Zhou et al., 2020). The increase in NLR reflects the depletion of lymphocytes and the increase in neutrophils that produce proinflammatory cytokines, and can predict the severity of clinical outcomes (Liu et al., 2020). Compared with females, males are more likely to exhibit systemic inflammation, ferritin, NLR>6, and a higher percentage of monocytes ​ (Qin L. et al., 2020). A recent study found that men have higher circulating innate inflammatory cytokines IL-8 and IL-18 by comparing the differences in immune response between male and female patients with COVID-19 (Takahashi et al., 2021). In addition, AR can increase the number and function of circulating neutrophils and increase the production of IL-1β, IL-10, IL-2, and transforming growth factor-β by immune cells (Klein and Flanagan, 2016). These cytokines are related to AKI caused by cytokine storm. D-dimer is usually elevated in patients with COVID-19. It is related to the severity of the disease and is also one of the indirect factors leading to AKI (Cui et al., 2020). Several studies have shown that although there is no significant difference in the expression level of D-dimer between male and female patients with COVID-19, the platelet count of males is significantly lower than that of females (Su W. et al., 2020; Yoshida et al., 2021). Males also have a higher risk of venous thromboembolism (VTE) than females (Baglin et al., 2004; Eichinger et al., 2010). Sepsis and endothelial dysfunction are also indirect factors that cause AKI. In general, females recover better than males from illnesses caused by infectious diseases, sepsis, trauma, or injury (McClelland and Smith, 2011; Markle and Fish, 2014). Immunity, inflammation, and hypercoagulability markers all have gender differences, and the biological factors underlying them are also deserve further investigation.

The baseline conditions of patients with COVID-19 at the time of admission also affect the occurrence of AKI. Early epidemiological reports indicated that hypertension, diabetes, cardiovascular disease, and CKD are risk factors for SARS-CoV-2 infection to cause AKI (Mesropian et al., 2016; Del Sole et al., 2020). Although epidemiology also showed that females have a higher prevalence of CKD than males (Murphy et al., 2016), lifetime risk studies have found that females may have slower kidney function decline compared with males (Albertus et al., 2016). Gender differences in the progression of CKD could be attributed to a variety of factors, including sex hormones, kidney hemodynamics, and differences in kidney quality between males and females (Neugarten, 2002; Neugarten and Golestaneh, 2013). Moreover, the frequency of increased indicators of myocardial injury in males during hospitalization is much higher than that in females, indicating that males are more susceptible to myocardial injury and heart failure after SARS-CoV-2 infection (Su W. et al., 2020). This condition impairs cardiac output and kidney perfusion, leading to gender differences in AKI.

By summarizing the relevant mechanisms and reasons, we provide a possible explanation for the high incidence of AKI in male patients with COVID-19. These mechanisms and data will help understand the gender differences in kidney injury caused by SARS-CoV-2 and design better prevention and treatment strategies.

## Ethnic Differences in the Incidence of AKI Among Patients With COVID-19

Current epidemiological data indicate that African Americans or blacks are more likely to be affected by SARS-CoV-2 and have worse outcomes (https://coronavirus.jhu.edu/data/racial-data-transparency). The U.S. National health statistics have documented extensive health disparities for Black patients with COVID-19, they suffer a threefold greater infection rate and a sixfold greater mortality rate than their white counterparts (Yancy, 2020). The high prevalence and poor health outcomes of blacks reflect a complex set of factors, including income, education history, and occupational differences (Smith et al., 2011). Recent data show that only 16% of Hispanics and 20% of Blacks can work from home, and they account for an excessively high proportion of essential roles that require in-person work, resulting in frequent and prolonged exposure to hazardous environments (Dorn et al., 2020). Many black communities reside in poverty areas with high housing density, high crime rates, and difficult access to healthy food. Low socioeconomic status is a risk factor for total mortality independent of other risk factors, and is related to the occurrence and development of a variety of underlying diseases, such as cardiovascular disease, diabetes, and CKD. It has been confirmed that the existence of underlying diseases could affect the infection rate and outcome of COVID-19 (Yancy, 2020). Furthermore, a large-scale survey found that black and Latinx respondents had a serious lack of understanding of the symptoms and transmission of COVID-19 (Alsan et al., 2020). These social determinants of health put minorities who live in high-risk communities at greater risk of disease, not just for the basic diseases but now for the COVID-19 infection rate and mortality rate.

The occurrence of AKI during COVID-19 infection is associated with high mortality. Data from China and the United States indicate that male sex, older age, Black race, diabetes, CKD, hypertension, cardiovascular disease, congestive heart failure, and higher body mass index (BMI) are associated with AKI in COVID-19 (Nadim et al., 2020). Several studies have shown that Black race is a risk factor for COVID-19-induced AKI. Nimkar et al. pointed out that among patients with COVID-19, older age, CKD, hyperlipidemia, and being of African American descent showed higher odds of AKI (Nimkar et al., 2020). Charoenngam et al. compared the hospital outcomes of Black and White hospitalized patients with COVID-19 at Boston Medical Center, the largest safety net hospital in New England. After adjusting for age, gender, BMI, potential type 2 diabetes, hypertension, and baseline estimated glomerular filtration rate (eGFR), the odds of AKI in black patients were statistically significantly higher (aOR 2.16, 95% CI, 1.57–2.97) (Charoenngam et al., 2021). In order to determine the risk factors related to the development of AKI in patients with COVID-19, Hirsch et al. found that black race was both an independent risk factor and an independent predictor for the occurrence of AKI through multivariate analysis (Hirsch et al., 2020). However, the differences in risk observed in patients with COVID-19 based on race may reflect disparities in social, economic, environmental, and other stressors, which may increase the risk of AKI and its adverse consequences. The complex interactions between various factors that affect health outcomes require a better and deeper understanding. Bowe et al. observed patients with COVID-19 in the Veterans Affairs (VA) health care system and found that blacks showed a strong association with AKI (1.9 times). Moreover, they pointed out that the percentage of Black race can explain the spatial and temporal changes in AKI rates (Bowe et al., 2021). The VA health care system is the largest nationally integrated health system in the United States, it aims to provide equitable access and reduce care variations, and has provided high-quality care many times (Asch et al., 2004). This research reduces the influence of socioeconomic factors on the results. In addition, Fisher et al. adjusted the age, gender, race/ethnicity, socioeconomic status, and neighborhood crowding of patients with COVID-19 and found that black race remained a significant risk for AKI. And they identified male sex, Black race, and older age as risk factors for development of AKI, regardless of COVID-19 status (Fisher et al., 2020). At present, the mechanism of the high incidence of AKI in black patients with COVID-19 is still unclear. By consulting relevant literature, we found that in addition to socioeconomic factors, the susceptibility of blacks to AKI may also be related to biological factors.

SARS-CoV-2 infection can cause collapsible glomerulopathy in individuals. The autopsy results of multiple studies described collapsing focal segmental glomerulosclerosis (FSGS) in patients with COVID-19, who developed rapid progressive renal function impairment (Minami et al., 2020; Wu et al., 2020; Akilesh et al., 2021). The increased risk of collapsing glomerulopathy in patients with COVID-19 is related to the high-risk apolipoprotein L1 (APOL1) genotype (G1, G2) (Larsen et al., 2020). The APOL1 risk genotypes were originally identified in African Americans with FSGS and/or ESKD, and FSGS is usually thought to arise from podocyte dysfunction (Genovese et al., 2010; Reidy et al., 2018). About 13% of African Americans have the APOL1 high-risk genotype, and these individuals have a 3- to 30-fold increased risk of various forms of kidney disease (Friedman and Pollak, 2020). Reports of collapsing FSGS related to SARS-CoV-2 infection are common among blacks. A study found that 6 of 7 COVID-19 patients with collapsing glomerulopathy were black ethnicity, and 1 black patient was found to carry the high-risk G1 genotype (Akilesh et al., 2021). By contrast, Su et al. reported an autopsy study of patients who died of COVID-19 with multiple organ complications in China. In this study, no patients developed collapsing glomerulopathy (Su H. et al., 2020). The association between high-risk APOL1 genotypes and kidney damage is highly important for understanding the ethnic differences in the onset of AKI in patients with COVID-19. APOL1 is a constituent of the high-density lipoprotein complexes and plays a vital role in lysing trypanosomes that cause African sleeping sickness (Albertus et al., 2016). The two coding variants of APOL1 (G1 and G2) are present at high frequency in individuals of recent African descent. These two genetic variants will cause amino acid changes, thereby changing the function of APOL1. Inheritance of two risk alleles will significantly increase the risk of kidney disease, including FSGS, ESKD caused by hypertension (Lipkowitz et al., 2013), HIV-associated nephropathy (Kopp et al., 2011; Kasembeli et al., 2015), lupus-associated kidney disease (Larsen et al., 2013; Freedman et al., 2014), and subtypes of membranous nephropathy (Larsen et al., 2014). However, individuals with the high-risk genotype of APOL1 do not universally suffer from kidney disease. Other factors must be needed to cause APOL1 nephropathy in high-risk groups. Such as viral infection, the link between viremia and APOL1 nephropathy also supports the idea that the virus can activate the APOL1 response to cause kidney damage (Divers et al., 2013; Freedman et al., 2018). In inflammatory settings, the expression of APOL1 is enhanced, and it has a strong up-regulation response to interferons, lipopolysaccharide, Toll-like receptor agonists, TNF, and other cytokines (Zhaorigetu et al., 2008; Nichols et al., 2015), which may contribute to a cytokine storm. In addition, a study of community-dwelling black adults found that patients with one or two risk alleles have a higher risk of sepsis than those carrying no APOL1 risk alleles (Chaudhary et al., 2019). As mentioned above, cytokine storm and sepsis are indirect factors of AKI caused by SARS-CoV-2 infection. These pieces of evidence provide a reasonable explanation for the ethnic differences in AKI among patients with COVID-19. If these mechanisms are confirmed, this phenomenon will have an important impact on public health.

Moreover, multiple studies have reported a higher prevalence of comorbidities, such as obesity, diabetes, hypertension, and CKD, in Black patients with COVID-19 (Chang et al., 2021; Wiley et al., 2021; Yoshida et al., 2021). The existence of comorbidities is a risk factor for the occurrence of AKI, which further explains why Black patients with COVID-19 suffer from AKI more than other races. The reason why Blacks are more affected by COVID-19 and have poor outcomes is also related to vitamin D deficiency. Vitamin D has been proven to be protective against COVID-19 infectivity and severity. It is known that there are significant ethnic differences in the genes encoding vitamin D-binding protein (DBP). Blacks are more likely to have variants of this gene, leading to low DBP levels and impaired vitamin D synthesis and metabolism (Nizamutdinov et al., 2019). In addition, the increase in melanin in black skin reduces the absorption of sunlight required to produce vitamin D (Nair and Maseeh, 2012; Cashman et al., 2016). Vitamin D deficiency is more common in individuals with obesity. Systematic reviews and meta-analysis showed a 35% higher prevalence of vitamin D deficiency in individuals with obesity (Pereira-Santos et al., 2015). Among patients with COVID-19, Blacks are more obese than other races. A study on the clinical aspects and outcome of COVID-19 in Black patients found that the average BMI of hospitalized patients is in the “obese” range, which is higher than the national average (Gupta et al., 2021). Vitamin D modulates the immune system by suppressing the T helper 1 immune profile and upregulating the expression of regulatory T cells, thereby reducing the severity of cytokine storm. Therefore, vitamin D deficiency puts Black patients at a higher risk of cytokine storm and resulting systemic and intrarenal inflammation (Charoenngam et al., 2021).

## Conclusion

**Figure 3:**
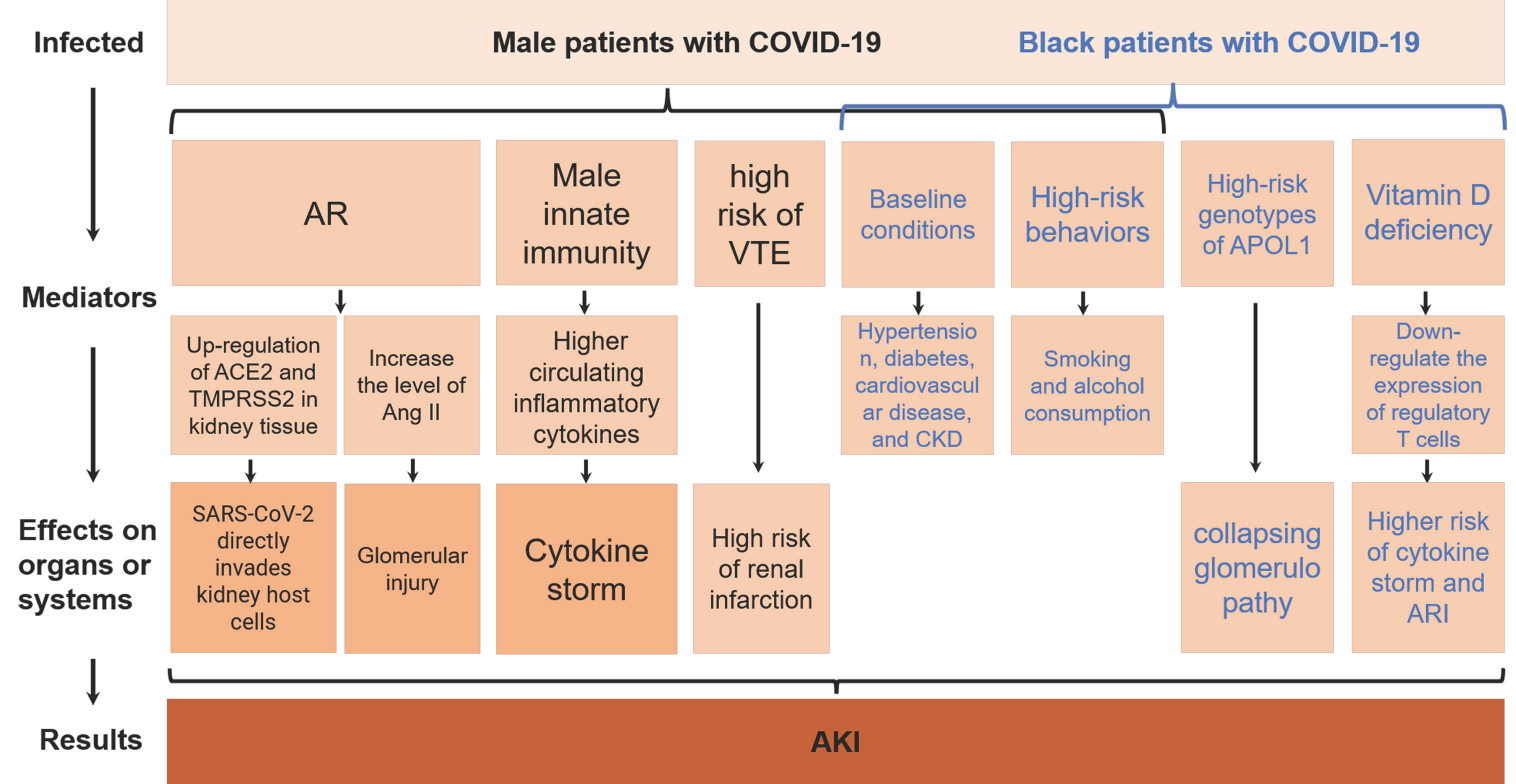
AKI, acute kidney injury; AR, androgens; SARS-CoV-2, Severe Acute Respiratory Syndrome Coronavirus 2; COVID-19, coronavirus disease 2019; ACE2, angiotensin-converting enzyme 2; TMPRSS2, type II transmembrane serine protease; Ang II, angiotensin II; VTE, venous thromboembolism; CKD, chronic kidney disease; APOL1, apolipoprotein L1; ARI, acute respiratory infection.

AKI is a serious complication of COVID-19 and an indicator of poor prognosis (Cheng et al., 2020a; Nadim et al., 2020; Varga et al., 2020). Therefore, it is very important to understand the gender and ethnic differences in the development of AKI in COVID-19 patients. Through literature search and analysis, we found that male and black races in COVID-19 patients are more likely to progress to AKI, and summarized the related mechanisms (**Figure 3**). The biological factors underlying these differences deserve further investigation. Proposing appropriate interventions based on these mechanisms is of great significance for improving the prognosis of COVID-19 patients.

## Author Contributions

WH, XL, and BH searched the literature and conceived and wrote the review. DL, LC, YL, KZ, YT, and SX revised the paper, tables and graphic abstract. GW and BF critically appraised the literature and made an intellectual contribution to the work. All authors read and approved the final manuscript.

## Funding

This study was supported by the National Natural Science Foundation of P.R. China (Grant Nos. 81560419, 81960512, and 81760457), Jiangxi Provincial “Double Thousand Plan” Fund Project (Grant No. jxsq2019201027), Key Project of Natural Science Foundation of Jiangxi Province (20212ACB206013), and Youth Project of Natural Science Foundation of Jiangxi Province (20212BAB216037).

## Conflict of Interest

The authors declare that the research was conducted in the absence of any commercial or financial relationships that could be construed as a potential conflict of interest.

## Publisher’s Note

All claims expressed in this article are solely those of the authors and do not necessarily represent those of their affiliated organizations, or those of the publisher, the editors and the reviewers. Any product that may be evaluated in this article, or claim that may be made by its manufacturer, is not guaranteed or endorsed by the publisher.
